# Classification of Literary Works: Fractality and Complexity of the Narrative, Essay, and Research Article

**DOI:** 10.3390/e22080904

**Published:** 2020-08-17

**Authors:** Aldo Ramirez-Arellano

**Affiliations:** Sección de Estudios de Posgrado e Investigación, Unidad Profesional Interdisciplinaria de Ingeniería y Ciencias Sociales y Administrativas, Instituto Politécnico Nacional, Ciudad de México 07738, Mexico; aramirezar@ipn.mx; Tel.: +52-5557296000

**Keywords:** complex networks, literary works, genre classification, stylistic attributes, lemmatization, renormalisation process

## Abstract

A complex network as an abstraction of a language system has attracted much attention during the last decade. Linguistic typological research using quantitative measures is a current research topic based on the complex network approach. This research aims at showing the node degree, betweenness, shortest path length, clustering coefficient, and nearest neighbourhoods’ degree, as well as more complex measures such as: the fractal dimension, the complexity of a given network, the Area Under Box-covering, and the Area Under the Robustness Curve. The literary works of Mexican writers were classify according to their genre. Precisely 87% of the full word co-occurrence networks were classified as a fractal. Also, empirical evidence is presented that supports the conjecture that lemmatisation of the original text is a renormalisation process of the networks that preserve their fractal property and reveal stylistic attributes by genre.

## 1. Introduction

A complex network as an abstraction of a language system has attracted attention in the last decade. The current linguistics research, based on the complex network approach, follows three major lines [1,2]: characterisation of human language as a multi-level system, linguistic typological research using quantitative measures, and the relationship between system-level complexity of human language and its microscopic features.

Word co-occurrence networks and their measures have been widely employed to analyse the syntactic features for multiple purposes, such as: identifying authors’ writing styles [3,4,5,6,7,8] and evaluating machine translations [9]. Also, Ferraz de Arruda, Nascimento Silva [10], as well as F. de Arruda, Q. Marinho [11] built a complex network where the nodes are the representation of adjacent paragraphs that share a minimum semantical content to classify the text as real (written by an author) or randomly constructed (built from random blocks of real texts).

In most of the research mentioned above, well-known measures such as: node degree (*k*), shortest path length (*spl*), betweenness (*b*), clustering coefficient (*cc*), and the average of nearest neighbourhoods’ degree (*nnd*) are applied to characterise the word co-occurrence networks. The *k*, *b*, and *nnd* are centrality measures that characterise local properties of the network that are useful for authorship attribution [3,4,5,6,7,8]. However, these measures do not capture the global network structure that could give us insight into the literary genre. This research aims at showing that local and global measures of the word co-occurrence networks—of literary works of Mexican writers—let us classify them according to the genre. Thus, the following research questions are formulated:

1. Are measures of the complex network useful to classify literary works by genre?
2. Is the full word co-occurrence network of literary works fractal?
3. Do pre-process tasks such as: deletion of number, punctuation, functional words, and lemmatisation generate fractal networks?

## 2. Measures of Complex Networks

Formally, a network is defined by *G = (V*, *E)* where *V* is the vertexes or nodes, and *E* is the edges. The complex networks exhibit non-trivial topological features that do not occur in simple networks, such as: lattices or random graphs [12], and their overall behaviour cannot be predicted by observing the behaviour of their nodes [13]. Since the complex network theory has its root in graph theory, some measures are presented below.

The degree of a node *i* is defined by:

(1) $$k_{i}=\sum_{j}^{N}v_{ij}$$

where *j* represents a given neighbour of the node *i*, and *N* is the total neighbours. The value of *v*_ij_ is defined as one, if there is a connection between nodes *i* and *j*, and as 0 otherwise.

Similarly, the betweenness of a node is defined as:

(2) $$b_{i}=\sum_{j,m\neq i}^{}\frac{L_{jm}(i)}{L_{jm}}$$

where *L_jm_*, is the number of shortest paths between nodes *j* and *k*, and *L_jm_(i)* is the shortest paths between nodes *j* and *m* that go through *i*.

The average nearest neighbourhoods’ degree (*nnd*) of a given node can be computed by:

(3) $$nnd_{i}=\sum_{j\in V(i)}^{}\frac{k_{j}}{k_{i}}$$

where *k_i_* is the degree of the node *i*, and the set *V(i)* contains its nearest neighbours, and *k_j_* is the degree of a given neighbour.

A definition of network clustering is expressed by:

(4) $$cc\left(G\right)=\frac{3\tau }{spl\left(2\right)}$$

where τ is the number of triangles of the network and *spl(2)* is the shortest path of length two. A “triangle” is a set of three nodes in which each contacts the other two.

### 2.1. Fractality of Complex Networks

A fractal is an object that is similar to itself on all scales [14]. A network is a fractal network if its box-covering follows the power law given by:

(5) $$N_{b}\left(l\right)∼\beta l^{-d_{b}}$$

where *N_b_(l)* is the minimum number of boxes of diameter *l* to cover the network—the procedure of box-covering that gives us this number is detailed later—*β* is the scaling factor, and *d_b_* is the box dimension of a complex network that can be obtained as follows:

(6) $$d_{b}=-\underset{l\to 0}{\lim}\frac{\ln N_{b}\left(l\right)}{\ln l}$$

On the other hand, a non-fractal network is characterised by a sharp decay of *N_b_(l)*, with *l* described by an exponential function as follows [15,16]:

(7) $$N_{b}\left(l\right)∼\beta e^{-d_{b}l}$$

### 2.2. Complexity of Networks

The complexity measure of a network proposed by Lei, Liu [17] is defined as:

(8) $$c\left(G\right)=d\left(G\right)s\left(G\right)$$

where $d\left(G\right)=\left|E\right|/\left(4CR^{3}/3\Delta \right)$ is the absolute density [18]; |*E*|, *C*, *R*, and Δ are the number of edges, circumference, radius, and diameter of the network, respectively. $s\left(G\right)=-k\sum_{i=1}^{\left|V\right|}\left(p_{i}^{q_{i}}-p_{i}\right)/\left(1-q_{i}\right)$ is known as structure entropy based on degree and betweenness [17], where *k* is the Boltzmann constant, |*V*| is the number of nodes, $p_{i}=\frac{k_{i}}{\sum_{i=1}^{\left|V\right|}k_{i}}$, $q_{i}=1+\left(b_{\max}-b_{i}\right)$, and *b_max_* is the maximum value of the betweenness computed by the Equation (2). This measure captures the topology of the networks, but it is not affected by scales and their types.

### 2.3. Box-Covering of Complex Networks

**Figure 1:**
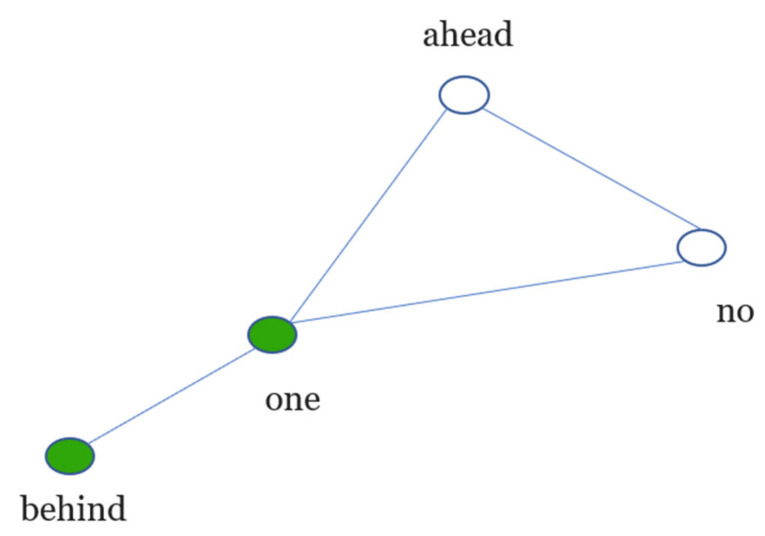
Word co-occurrence network of “No one behind, no one ahead”; the nodes in same colour belong to the same box.

To obtain *N_b_(l)*, consider the phrase “No one behind, no one ahead”. Its word co-occurrence network is shown in Figure 1. The number of boxes to cover the network *N_b_(l)* for *l = 1*, and *l* = Δ + 1—where Δ is the diameter of the network—is the number of nodes of the network and one, respectively. The *N_b_(l)* from 2 to Δ is not a trivial answer.

**Figure 2:**
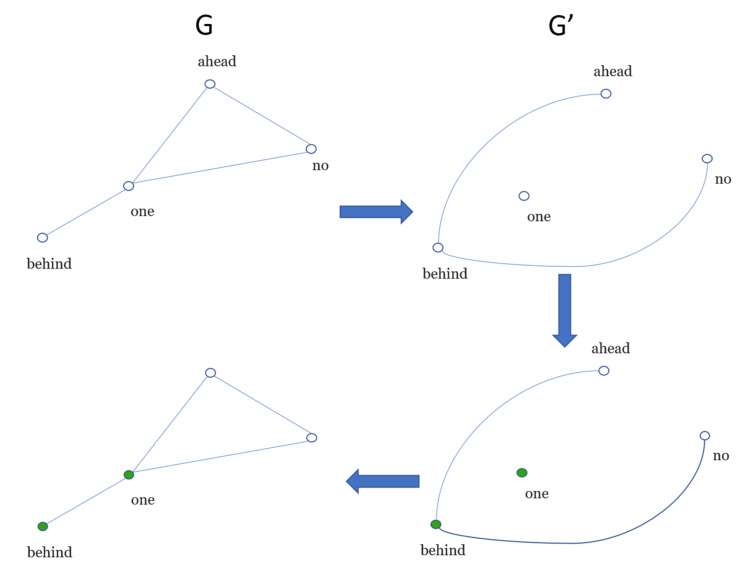
Covering of the network for a given box size (*l* = 2). The number of boxes in this network is *N_b_(*2*)* = 2.

For example, *N_b_(l = 1) = 4 and N_b_(l* = Δ *+* 1*)* = 1 for the network of Figure 1. To obtain the *N_b_(l = 2),* we first compute a dual network (*G’*) from the original (*G*) as follows: given a distance *l;* two nodes *i*, *j*, in the dual network, are connected if the distance between *l_ij_* is greater than or equal to *l*. For example, we start the procedure from the node “no”, see Figure 2; “no” and “behind” have a distance of two in *G,* thus, they will be connected in *G’*. Next, the node “ahead” as the starting node is chosen—notice that the distance from it to “behind” is two—thus, a connection in *G’* will be drawn (see Figure 2).

Then, the nodes of *G*’ must be coloured following a single rule: two nodes directly connected will be painted different colours. The nodes of the resulting coloured dual network *G*’ are mapped to original network *G*. The number of colours of *G*’ represents the minimum number of boxes *N_b_*(l) of a given value of *l* to cover the network. The nodes of *G*, in the same colour, belong to the same box. The procedure described above is repeated until *l* = Δ + 1. For more profound details of the box-covering algorithm, the reader is referred to the work of Chaoming, Lazaros [19]. Since *l* vs. *N_b_*(l) characterises the topology of the network, the area under the box-covering curve, *l* vs. *N_b_*(l) (AUB), was also included in the measures of the word co-occurrence network.

### 2.4. Robustness of Fractal Networks

Intentional network attacks are based on different centrality measures such as: the node degree or betweenness. They differ in the approach to compute those centrality measures such as: computing the global degree or betweenness, then performing the attack, or recomputing the centrality measure after a node is removed [20,21,22,23]. The fraction of nodes necessary to break down a fractal network (*p_c_*) by a random attack are close to the total number of nodes; thus, these networks are extremely robust [24]. On the other hand, this robustness decreases drastically when the nodes with a high degree are selected to be removed [20,25]. This vulnerability to intentional attack relies on that a few nodes, with a high degree, maintain the connectivity of the network [26]. The robustness of each network is quantified by the size of the largest connected component *C_c_* after removing a fraction *p* node from the network [20,24,26,27] when $C_{c}\left(p_{c}\right)≃0$ the network has been disintegrated. The value of *p_c_* is low for fragile networks, and the opposite for robust networks.

**Figure 3:**
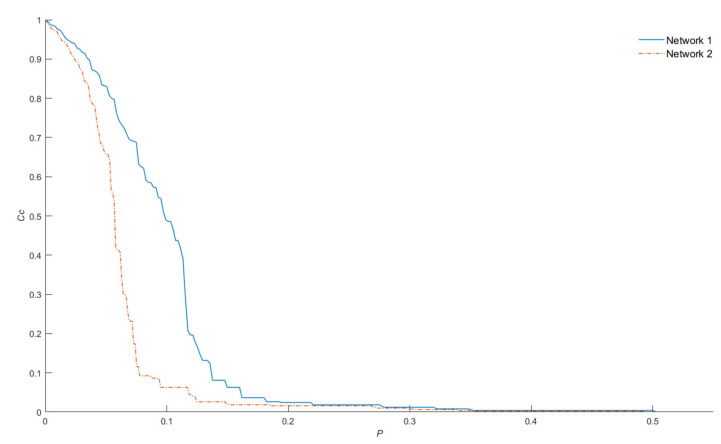
The damage of an individual node removal of network one and two. Although the *p_c_* of networks one and two are 0.5 and 0.49, respectively, the area under the robustness curve reflects more precisely the vulnerability of the networks (0.0956 for network one and 0.060 for network 2).

Although the *p_c_* value is useful for measuring the overall damage caused by the attack strategy, it does not reflect the damage of an individual node removal; for example, Figure 3 shows the plot of *C_c_* vs. *p*, where the value of *p_c_* is 0.5 and 0.49 for networks one and two, respectively.

This means that for both networks, it is necessary to remove approximately 50% of the nodes to disintegrate them in components that contain at most one node. Moreover, based on Figure 3, the removal of the nodes from network two causes more damage than the removal of those from network one. This damage can be quantified by computing the Area Under the Robustness Curve (AURC)—0.0956 for network one and 0.060 for network 2—to a higher the value, the higher the robustness of the network. The AURC of the attack performed by node degree was included as a measure of network robustness instead of *p_c_.*

## 3. Materials and Methods

From seven Mexican writers —Juan José Arreola Zúñiga, Carlos Fuentes Macías, Jorge Ibargüengoitia Antillón, Carlos Monsiváis Aceves, José Emilio Pacheco Berny, Octavio Irineo Paz Lozano, and Alfonso Reyes Ocha—21 essays, 21 narratives (15 tales and six novels), and 21 research articles were the corpus for this research (see Table 1). Noticeably, some authors wrote titles classified as essays, tales, or novels, such as Carlos Fuentes, Jorge Ibargüengoitia, and José Emilio Pacheco. The essays, narratives, and research articles were published between 1911 and 2019. All the titles were obtained in an electronic format such as pdf and then converted to plain text.

The node degree (*k*), betweenness (*b*), shortest path length (*spl*), clustering coefficient (*cc*), and nearest neighbourhoods’ degree (*nnd*), as well as more complex measures such as: the fractal dimension (*d_b_*) obtained by the Equation (6), the complexity of a network *c(G)* given by the Equation (8), the Area Under Box-covering (*AUB*), and the Area Under the Robustness Curve (AURC), were computed for each network of each title. Statistical analysis was carried out to select those measures that have a significant difference by literary genres and produce a better classification.

Then the Support Vector Machine (SVM), Naïve Bayes (NB), Decision Tree (DT), and Neural Network (NN) implemented in Weka [28] and fourth data mining views—described later and based on the measures mentioned above—were employed to classify the literary works. The hyperparameter optimisation of the data mining techniques was conducted by sequential model-based algorithm configuration [29,30]. The hidden layers and the nodes learning function of NN were 28 and sigmoid, respectively. The polynomial kernel was used in SVM, and all measures of the networks were normalised before training and validating SVM and NN. The NN technique was used with a normal distribution to estimate the probabilities of the network measures. DT uses the C4.5 algorithm [31].

**Figure 4:**
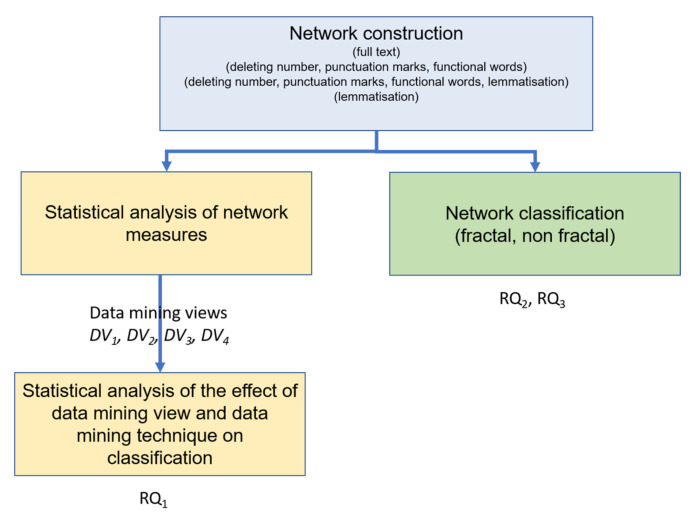
The experimental design followed to answer the research questions.

The efficacy of each data mining technique and data mining views was validated by 5-fold cross-validation, comparing the Area under the Receiver Operating characteristic Curve (AROC). The AROC is useful to measure the performance of a data mining technique when the dataset is unbalanced [32]. Values of AROC closer to 1 mean a better classification than those closer to 0.5. This analysis shows the impact of data mining techniques and the measures on the classification of literary works. These results answer research question one (see Figure 4). Also, the accuracy of classification is presented as additional information that is computed as (TP Positive (TP) + False Positive (FP) + False Negative (FN) + True Negative (TN)). The computation of AROC and accuracy are well-known for a two-class problem. Furthermore, for a multi-class problem, for each time one class could be considered as positive, then all the others as negative. This means that TP, TN, FP, and FN are calculated for each class. Therefore, a confusion matrix and AROC curve is obtained for each class (see [33,34] for more details).

A set of word co-occurrence networks of each title was obtained and the first network was built using the full text. The second was obtained by deleting numbers and functional words. A lemmatisation stage created the third after numbers and functional words deletion, and the fourth network was attained only through a lemmatisation stage (see Figure 4).

The networks were obtained by using the full text, by deleting numbers and functional words, by adding a lemmatisation stage after the numbers and functional words deletion, and through only a lemmatisation stage, are classified as fractal or non-fractal. Thus, research question two and three will be answered.

## 4. Results and Discussion

Table 2, Table 3, Table 4 and Table 5 show the descriptive statistics by literary genre of the three types of networks—the first was built using the full text; the second was built by deleting numbers, punctuation marks, and functional words; the third was built by adding a lemmatisation stage; and the fourth was built through only a lemmatisation stage, denoted by subscripts *f, nf*, *l* and *ol*, respectively.

An Analysis of Variance (ANOVA) or a Kruskal–Wallis test—an ANOVA test carried out if the normality and homoscedasticity assumptions were valid for the given measure—was performed to select the measures of complex network that are influenced by essay, tale, novel, and research article genres. The one-way ANOVA conducted on the individual influence of essay, tale, novel, and research article on *k*_c_, *spl_f_*, *cc_f_*_,_ *d_bf_*, *c(G)_f,_* and *AURC_f_* shows significant effects: *F*(3,59) = 12.81, *p* < 0.0001; *F*(3,59) = 15.039, *p* < 0.0001; *F*(3,59) = 14.77, *p* < 0.0001; *F*(3,59) = 19.27, *p* < 0.0001; *F*(3,59) = 6.40, *p* < 0.001; and *F*(3,59) = 22.35, *p* < 0.0001. Similarly, a Kruskal–Wallis test shows a significant difference of the literary genres on *nnd_f_*, *AUB_f_*, *b_f_*; *H(*3*)* = 29.44, *p* < 0.0001; *H (*3*)* = 27.98, *p* < 0.0001; and *H(*3*)* = 28.68, *p* < 0.0001.

The one-way ANOVA conducted on the individual influence of essay, tale, novel, and research article on *spl_nf_*, *cc_nf_*_,_ *d_bnf_*, and *AURC_nf_* shows significant effects: *F*(3,59) = 3.70, *p* = 0.016; *F*(3,59) = 6.17, *p* = 0.001; *F*(3,59) = 3.00, *p* ≤ 0.037; and *F*(3,59) = 4.28, *p* = 0.008. On the other hand, no effect on *k_nf_ F*(3,59) = 2.65, *p* = 0.057; *nnd_nf_ F*(3,59) = 1.12, *p* = 0.347; *b_nf_ F*(3,59) = 0.227, *p* = 0.877; *c(G)_nf_ H(*3*)* = 3.99, *p* = 0.262; and *AUB_nf_ H(*3*)* = 1.29, *p* = 0.731 by genres were found. Although *spl_nf_*_,_ *cc_nf_*_,_ *d_bnf_*, and *AURC_nf_* have a significant difference, they do not provide additional information—of those provided by the measures of full-text networks—to differentiate the genre. For example, *spl_nf_* is only statistically different for the novel and tale (see Table 6). However, *slp_f_* is statistically different for the novel, essay, and both the research article and tale. Thus, *spl_nf_*_,_ *cc_nf_*_,_ and *d_bnf_* were not included in the set of measures to build data mining models. Table 6 summarises the significant statistical difference for *spl_f_* and *spl_nf_*.

Finally, the one-way ANOVA conducted on the individual influence of essay, tale, novel, and research article on *spl_l_* and *AURC_l_* shows significant effects: *F*(3,59) = 17.62, *p* < 0.0001; *F*(3,59) = 4.28, *p* = 0.008. Similarly, a Kruskal–Wallis test shows a significant difference of *k_l_*, *cc_l_*_,_ *d_bl_*, *c(G)_l_, nnd_l_*, *AUB_l_*, and *b_l_* by genre: *H(*3*)* = 32.98.44, *p* < 0.0001; *H(*3*)* = 23.38, *p* < 0.0001; *H(*3*)* = 30.20, *p* < 0.0001; *H(*3*)* = 22.03, *p* < 0.0001; *H(*3*)* = 29.38, *p* < 0.0001; *H(*3*)* = 32.40, *p* < 0.0001, *p* < 0.0001; and *H(*3*)* = 32.64, *p* < 0.0001.

**Figure 5:**
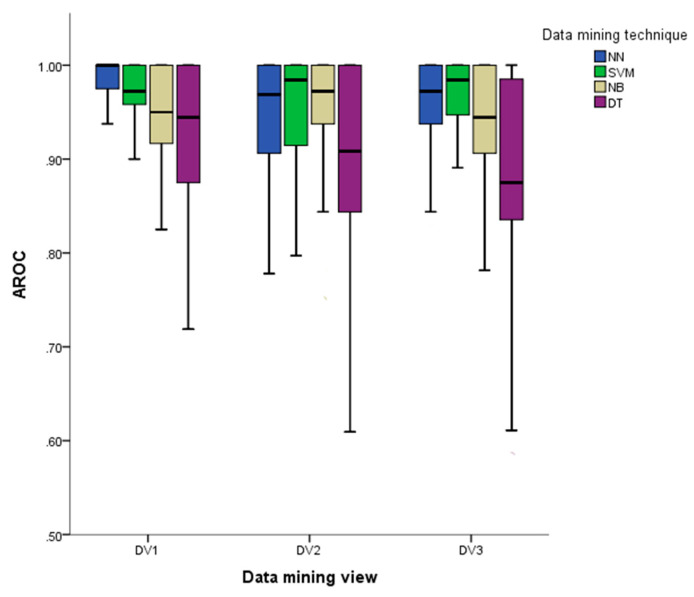
The effect of data mining view and data mining technique on Area under the Receiver Operating characteristic Curve (AROC).

After these analyses, the *spl_f_*, *k_f_*, *nnd_f_*, *cc_f_*, *b_f_*, *db_f_*, *AURC_f_*, *AUB_f_*, *c(G)* *_f_*, *spl_l_, k_l_, nnd_l_*, *cc_l_*, *b_l_*, *db_l_*, *AURC_l_*, *AUB_l_*, and *c(G)_l_* were selected to classify the genre of each literary work. This set of measures is a data mining view named *DV_1_*, and *DV_1_* was compared with a data mining view named *DV_2_* that contains all the measures computed on the three types of co-occurrence networks described previously. Also, a third data mining view named *DV_3_*, which contains only the measures *spl, k, nnd, cc,* and *b* obtained from the three types of co-occurrence networks, was tested to show that measures such as *d_b_*, *c(G)*, *AUB*, and *AURC* contribute to capturing the features of the literary genre. Since the influences of the data mining technique and data mining view on the AROC need to be tested, a two-way ANOVA is appropriate for this purpose, providing the data is normal and homoscedastic [32,35]. However, the AROC generated by our experiments does not meet these assumptions; thus, a Scheirer–Ray–Hare test [36,37] was used instead. A Scheirer–Ray–Hare test shows there is a significant difference among the AROC of the data mining views: *H*(2) = 21.496, *p <* 0.001, the data mining techniques: *H*(3) = 84.79, *p <* 0.001, and the interaction between both: *H*(6) = 30.167, *p <* 0.001. Figure 5 summarises the effect of both data mining view and data mining technique on AROC that are detailed below.

A Kruskal–Wallis test shows that *DV_1_*, *DV_2_*, and *DV_3_* affect the median of the AROC:

*H* (2) = 21.496, *p* < 0.001. A posthoc Mann–Whitney test using a Dunn–Sidak adjustment [38] (α = 0.0169) shows that the median of *DV_1_* (*Mdn* = 0.975) is higher than *DV_2_* (*Mdn* = 0.968)—*U* (N_Dv1_ = 400, N_Dv2_ = 400) = 68704, *z* = −3.59, *p* < 0.001 and *DV_3_* (*Mdn* = 0.955)—*U* (N_Dv1_ = 400, N_Dv2_ = 400) = 66131, *z* = −4.388, *p* < 0.001. Thus, the statistical analysis carried out on the measures of three types of networks is useful to select relevant measures that increase the AROC. No statistical difference was found between *DV_2_* and *DV_3_*, *U* (N_Dv2_ = 400, N_Dv2_ = 400) = 78117, *z* = −0.59, *p* = 0.236. This evidence suggests that well-known measures such as: node degree, shortest path length, betweenness, clustering coefficient, and the average of nearest neighbourhoods’ degree—used to build *DV_3_*—applied in the previous research to identify authors’ writing styles [3,4,5,6,7,8] are not enough to produce a higher AROC. On the other hand, more complex measures such as: *d_b_*, *c(G)*, *AUB*, and *AURC* improve the classification.

Similarly, the Kruskal–Wallis test shows that the medians of the AROC obtained from NN, SVM, NB, and DT affect the AROC, *H* (3) = 84.793. A posthoc Mann–Whitney test using a Dunn–Sidak adjustment [38] (α = 0.0085) shows that the median of both NN (*Mdn* = 1.00) and SVM (*Mdn* = 0.975) were higher than those of NB (*Mdn* = 0.968)—see the corresponding row and column of Table 7 for the result of the pair-wise test e.g., row NB and column NN show a significant difference: *U* (N_NN_ = 300, N_NB_ = 300) = 36784.5, *z* = −3.995, *p* < 0.0001— and DT (*Mdn* = 0.911). No statistical difference between NN (*Mdn* = 1.00) and SVM (*Mdn* = 0.975) was found.

Then a significant difference between NB (*Mdn* = 0.968) and DT (*Mdn* = 0.911), was found. These results suggest that the *DV_1_* and the use of NN or SVM produce statistically equal values of AROC. The accuracy of NN and SVM based on *DV_1_* are 0.93 and 0.90, respectively, based on *DV_1_*.

To support the conjecture that deleting number, punctuation, and functional words do not have a significant effect on the AROC, the models of NN based on *DV_1_* and the fourth data mining view named *DV_4_*, which contain the measures from the networks built using the full text (*spl_f_*, *k_f_*, *nnd_f_*, *cc_f_*, *b_f_*, *db_f_*, *AURC_f_*, *AUB_f_*, and *c*(*G*) *_f_*) and those from networks built using only a lemmatisation stage (*spl_ol_, k_ol_, nnd_ol_, cc_ol_, b_ol_, db_ol_, AURC_ol_, AUB_ol_,* and *c(G) _ol_*), were compared. The Mann–Whitney test shows no statistical difference—*U* (*N_DV1_* = 100, *N*_DV2_ = 100) = 4793, *z* = −0.631, *p* = 0.528—between the AROC of *DV_1_* (*Mdn* = 1) and *DV_4_* (*Mdn* = 0.98). The accuracy of *DV_1_* and *DV_4_* is 0.93 for both. Thus, the deletion of the number and punctuation marks is not useful to reveal stylistic attributes by genre as lemmatisation does. Furthermore, all these stages together modify the network fractality, as the evidence presented later suggests. The accuracy of the NN model based on *DV_1_*, *DV_2_*, *DV_3_*, and *DV_4_* are 0.93, 0.90, 0.89, and 0.93, respectively.

To classify each network as fractal or non-fractal, the Akaike Information Criterion (AIC) [39] were computed for the networks based on the full text. The second network was obtained by deleting numbers, punctuation marks, and functional words. The third was created by adding a lemmatisation stage, and the fourth was attained only through a lemmatisation. The AIC is useful to classify networks as fractal and non-fractal [40]. To select the better mathematical model, first the AIC for power (denoted by subscript P) and exponential (denoted by subscript E) models—Equations (5) and (7)—were computed, then the minimum value is chosen (AIC_min_). ΔAIC_i_ was computed by AIC_i_ - AIC_min_, where *i* is the AIC of power or exponential models. The AIC’s rule of thumb is that the two models are statistically different if ΔAIC is greater than two, thus, the model with ΔAIC = 0 should be selected [41,42]. Table S2 of the supplementary material shows that the difference between ΔAIC_P_ and ΔAIC_E_ for about 87% of the full word co-occurrence network is higher than two; thus, the mathematical model for the relation *l* vs. *N_b_(l)* computed by the box-covering algorithm of these networks is the power model (see Equation (5)). Although for 13% of the networks, a model cannot be selected feasibly based on ΔAIC, the power model obtained the least value. Thus, most of the full word co-occurrence networks of literary works are fractal. This result supports the fractality founded in other languages and English literature by different mathematical analyses [43,44,45,46]. Noticeably, selecting the better model based on the adjusted coefficient of determination ($\begin{array}{c} {\bar{R}}^{2} \end{array}$) is rather difficult.

Similarly, Table S3 of the supplementary material shows that the difference between ΔAIC_P_ and ΔAIC_E_ for about 89% of the word co-occurrence networks—built by deleting numbers, punctuation marks, and functional words—suggests they are fractal; 2% were classified as exponential, and 9% were undetermined (since ΔAIC ≤ 2). However, adding a lemmatisation stage to the previous ones dilutes the fractality (25.3% are fractal, 33.3% are exponential, and 41.3 are undetermined), see Table S4. The lemmatisation stage alone preserves the fractality of the full-text networks (87% are fractals, and 13% are undetermined); see Tables S2 and S5, which show no difference between the AROC curve of the classification of literary works according to their genre. Note that the lemmatisation stage preserves the original fractality of the networks. Thus, this supports the conjecture that lemmatisation is a kind of renormalisation of a complex network that preserves the fractality. This paves the way to compare this linguistic renormalisation with that introduced by Song, Havlin [16].

## 5. Conclusions

This research aims at showing that measures of the word co-occurrence network of literary works—by Mexican writers—classifies them according to the literary genre. The local measures—such as: node degree, the average of nearest neighbourhoods’ degree, and global measures using shortest path length, betweenness, clustering coefficient, and the average of nearest neighbourhoods’ degree—widely used in the previous research to identify authors’ writing styles, produces acceptable values of AROC classification. However, more elaborate measures using fractal dimension, complexity, the AUB, and the AURC show an improvement of AROC. These measures capture the topology based on the minimum number of boxes to cover the network, the robustness, and the complexity measured by structural entropy and density. Precisely 87% of the full word co-occurrence networks were classified as a fractal. Thus, those findings support the conjecture that fractality occurs in the literary works of Mexican writers, as was previously reported by their English-speaking counterparts. Also, the empirical evidence suggests that the lemmatisation of literary works is a renormalisation stage that preserves the original text fractality. On the contrary, the deletion of numbers, punctuation marks, and functional works, as well as lemmatisation, dilute the fractality. The number of literary works included in this study limit the generalisation of this conjecture. Also, it would be interesting for future research directions to compare the renormalisation induced by a lemmatisation stage—linguistic renormalisation—to renormalisation of networks based on the box-covering algorithm.

## Figures and Tables

**Table 1:** The genre, number of titles, and primary author of the corpus.

Genre	Number of Titles	Primary Author
Essay	6	Alfonso Reyes Ochoa
Essay	3	Carlos Fuentes Macías
Essay	6	Carlos Monsiváis Aceves
Essay	6	Octavio Irineo Paz Lozano
Narrative (Tale)	2	Carlos Fuentes Macías
Narrative (Tale)	5	José Emilio Pacheco Berny
Narrative (Tale)	3	Jorge Ibargüengoitia Antillón
Narrative (Tale)	5	Juan José Arreola Zúñiga
Narrative (Novel)	1	Carlos Fuentes Macías
Narrative (Novel)	1	José Emilio Pacheco Berny
Narrative (Novel)	3	Jorge Ibargüengoitia Antillón
Narrative (Novel)	1	Juan José Arreola Zúñiga
Research Article	16	Several authors

**Table 2:** Mean and standard deviation by genre of node degree (*k*), betweenness (*b*), shortest path length (*spl*), clustering coefficient (*cc*), nearest neighbourhoods’ degree (*nnd*), fractal dimension (*d_b_*), complexity *c(G)*, the Area Under Box-covering (*AUB*), and the Area Under the Robustness Curve (*AURC*) of the networks built using the full text.

Genre	*K_f_(µ*–*σ)*	*b_f_ (µ*–*σ)*	*slp_f_ (µ*–*σ)*	*cc_f_ (µ*–*σ)*	*nnd_f_ (µ*–*σ)*	*d_bf_ (µ*–*σ)*	*c(G) _f_ (µ*–*σ)*	*AUB_f_ (µ*–*σ)*	*AURC_f_ (µ*–*σ)*
Essay	5.39–0.94	8249.16–4869.58	2.9–0.81	0.427–0.107	282.51–234.12	6.07–0.772	7.84 × 10^−4^–3.83 × 10^−4^	1510.61–969.37	0.0154–0.0031
Narrative (Tale)	4.76–0.536	3868.33–1709.2	3.02–0.94	0.311–0.082	99.57–55.59	5.06–0.801	7.22 × 10^−4^–3.62 × 10^−4^	685.86–315.90	0.0231–0.0067
Narrative (Novel)	6.81–1.01	13667.19–4796.52	2.78–0.061	0.577–0.088	559.34–233.84	7.42–0.644	6.78 × 10^−4^–2.48 × 10^−4^	2631.5–1013.36	0.01456–0.0017
Research Article	5.80–0.94	5995.1–2013.42	3.00–0.092	0.374–0.065	153.31–73.31	5.43–0.539	3.72 × 10^−4^–2.39 × 10^−4^	1068.4–379.76	0.0284–0.0071

**Table 3:** Mean and standard deviation by genre of node degree (*k*), betweenness (*b*), shortest path length (*spl*), clustering coefficient (*cc*), nearest neighbourhoods’ degree (*nnd*), fractal dimension (*d_b_*), complexity *c(G)*, the Area Under Box-covering (*AUB*), and the Area Under the Robustness Curve (*AURC*) of the networks built by deleting numbers and functional words.

Genre	*k_nf_(µ*–*σ)*	*b_nf_ (µ*–*σ)*	*slp_nf_ (µ*–*σ)*	*cc_nf_ (µ*–*σ)*	*nnd_nf_ (µ*–*σ)*	*d_bnf_ (µ*–*σ)*	*c(G)_nf_ (µ*–*σ)*	*AUB_nf_ (µ*–*σ)*	*AURC_nf_ (µ*–*σ)*
Essay	3.763–1.067	15092.572–8418.110	5.370–0.911	0.052–0.037	12.101–9.638	2.057–0.352	1.5 × 10^−5^–1.32 × 10^−5^	2299.330–1416.600	0.077–0.010
Narrative (Tale)	3.998–0.861	13572.998–7935.093	5.109–0.922	0.710–0.042	12.319–5.054	2.141–0.302	2.1 × 10^−5^–1.61 × 10^−5^	2022.467–1173.400	0.083–0.018
Narrative (Novel)	4.703–0.425	16327.442–11950.302	4.460–0.249	0.097–0.029	17.797–5.444	2.390–0.160	1.8 × 10^−5^–0.77 × 10^−5^	2656.333–1971.806	0.087–0.006
Research Article	3.483–1.054	15538.651–7392.827	5.770–1.03	0.037–0.024	10.648–9.539	1.940–0.383	1.2 × 10^−5^–0.59 × 10^−5^	2339.476–1314.540	0.070–0.012

**Table 4:** Mean and standard deviation by genre of node degree (*k*), betweenness (*b*), shortest path length (*spl*), clustering coefficient (*cc*), nearest neighbourhoods’ degree (*nnd*), fractal dimension (*d_b_*), complexity *c(G)*, the Area Under Box-covering (*AUB*), and the Area Under the Robustness Curve (*AURC*) of the networks built by deleting numbers, functional words, and lemmatisation stage.

Genre	*k_l_(µ*–*σ)*	*b_l_ (µ*–*σ)*	*slp_l_ (µ*–*σ)*	*cc_l_ (µ*–*σ)*	*nnd_l_ (µ–σ)*	*d_bl_ (µ–σ)*	*c(G)_l_ (µ*–*σ)*	*AUB_l_ (µ–σ)*	*AURC_l_ (µ–σ)*
Essay	4.081–1.649	21871.411–7787.529	5.418–0.960	0.009–0.005	9.074–7.389	1.990–0.358	1.37 × 10^−5^–1.87 × 10^−5^	2202.024–752.531	0.115–0.023
Narrative (Tale)	3.148–0.544	9538.206–4171.808	5.823–0.816	0.008–0.004	5.652–2.372	1.758–0.193	2.1 × 10^−5^–1.61 × 10^−5^	1121.733–411.3845	0.003–0.0149
Narrative (Novel)	6.197–1.796	25284.524–7739.246	4.181–0.499	0.020–0.010	20.689–10.965	2.489–0.379	1.9 × 10^−5^–1.49 × 10^−5^	2760.667–677.608	0.138–0.017
Research Article	4.849–0.691	11758.340–6325.385	4.296–0.352	0.207–0.010	11.169–2.802	2.234–0.155	2.8 × 10^−5^–1.85 × 10^−5^	1272.857–540.06	0.138–0.016

**Table 5:** Mean, and standard deviation by genre of node degree (*k*), betweenness (*b*), shortest path length (*spl*), clustering coefficient (*cc*), nearest neighbourhoods’ degree (*nnd*), fractal dimension (*d_b_*), complexity *c(G)*, the Area Under Box-covering (*AUB*), and the Area Under the Robustness Curve (*AURC*) of the networks built only by lemmatisation stage.

Genre	*k_ol_(µ–σ)*	*b_ol_ (µ–σ)*	*slp_ol_ (µ–σ)*	*cc_ol_ (µ–σ)*	*nnd_ol_ (µ–σ)*	*d_bol_ (µ–σ)*	*c(G)_ol_ (µ–σ)*	*AUB_ol_ (µ–σ)*	*AURC_ol_ (µ–σ)*
Essay	5.377–0.94	2943.853–1852.075	2.915–0.081	0.413– 0.093	280.854–232.41	6.041–0.775	7.85 × 10^−4^–3.84 × 10–^4^	1520.381–972.673	0.0151–.003
Narrative (Tale)	4.756–0.534	1324.018– 628.419	3.033–0.095	0.289– 0.06	99.135– 55.332	5.019–0.798	7.24 × 10^−4^– 3.63 × 10^−4^	693.8– 316.46	0.0242–0.007
Narrative (Novel)	6.794–1.014	5035.632–1864.014	2.793–0.062	0.507–0.093	555.98– 231.929	7.383–0.673	6.79 × 10^−4^–2.49 × 10^−4^	2644.833– 1012.555	0.0151–0.002
Research Article	5.777–0.466	2096.629–725.505	3.015–0.095	0.349–0.05	152.065–72.851	5.378–0.559	3.59 × 10^−4^–2.42 × 10^−4^	1078.357–379.765	0.0283–0.007

**Table 6:** The subsets built using the significant statistical differences between *slp_f_* and *slp_nf_* induced by the novel, essay, research article, and tale. The value in the intersection of each row and column is the means of each measure for a given genre.

Genre	Subset 1	Subset 2	Subset 3
Novel–*slp_f_*	2.78		
Essay–*slp_f_*		2.90	
Research Article–*slp_f_*			3.00
Tale–*slp_f_*			3.02
Novel–*slp_nf_*	4.46		
Essay–*slp_nf_*	5.10	5.10	
Research Article–*slp_nf_*	5.37	5.37	
Tale–*slp_nf_*		5.77	

**Table 7:** Pair-wise Mann–Whitney test using a Dunn–Sidak adjustment (α = 0.0085) among data mining techniques. The intersection of a row and a column presents the result of the test between the two data mining techniques.

	NN	SVM	NB	DT
NN	—			
SVM	*U* (N_NN_ = 300, N_SVM_ = 300) = 41859, *z* = −1.55, *p* < 0.120	—		
NB	*U* (N_NN_ = 300, N_NB_ = 300) = 36784.5, *z* = −3.995, *p* < 0.0001	*U* (N_SVM_ = 300, N_NB_ = 300) = 35311, *z* = −4.749, *p* < 0.0001	—	
DT	*U* (N_NN_ = 300, N_DT_ = 300) = 29119, *z* = −7.816, *p* < 0.0001	*U* (N_NN_ = 300, N_DT_ = 300) = 30523, *z* = −6.989, *p* < 0.0001	*U* (N_NN_ = 300, N_DT_ = 300) = 35438.5, *z* = −4.588, *p* < 0.0001,	—

## Supplementary Materials

The following are available online at https://www.mdpi.com/1099-4300/22/8/904/s1, Table S1 Title, primary author and the gender of the literacy works, Table S2, The adjusted determination coefficient, AIC, ΔAIC and classification for the networks based the full text, Table S3 The adjusted determination coefficient $\begin{array}{c} {\bar{R}}^{2} \end{array}$, AIC, ΔAIC and classification for the networks obtained by deleting numbers, punctuation marks and functional words, Table S4 The adjusted determination coefficient $\begin{array}{c} {\bar{R}}^{2} \end{array}$, AIC, ΔAIC and classification for the networks obtained by deleting numbers, punctuation marks, functional words and a lemmatisation stage, and Table S5 The adjusted determination coefficient, AIC, ΔAIC and classification for the networks obtained only by a lemmatisation stage.

## References

[1] Fang Y., Wang Y. (2018). Quantitative Linguistic Research of Contemporary Chinese. J. Quant. Linguist..

[2] Cong J., Liu H. (2014). Approaching human language with complex networks. Phys. Life Rev..

[3] Amancio D.R., Oliveira O.N., Costa L.d.F. (2012). Structure–semantics interplay in complex networks and its effects on the predictability of similarity in texts. Phys. A Stat. Mech. Appl..

[4] Akimushkin C., Amancio D.R., Oliveira O.N. (2018). On the role of words in the network structure of texts: Application to authorship attribution. Phys. A Stat. Mech. Appl..

[5] Mehri A., Darooneh A.H., Shariati A. (2012). The complex networks approach for authorship attribution of books. Phys. A Stat. Mech. Appl..

[6] Darooneh A.H., Shariati A. (2014). Metrics for evaluation of the author’s writing styles: Who is the best? Chaos Interdiscip. J. Nonlinear Sci..

[7] Machicao J., Corrêa Jr E.A., Miranda G.H., Amancio D.R., Bruno O.M. (2018). Authorship attribution based on Life-Like Network Automata. PLoS ONE.

[8] Stanisz T., Kwapień J., Drożdż S. (2019). Linguistic data mining with complex networks: A stylometric-oriented approach. Inf. Sci..

[9] Amancio D.R., Nunes M.D., Oliveira Jr O.N., Pardo T.A., Antiqueira L., Costa L.D. (2011). Using metrics from complex networks to evaluate machine translation. Phys. A Stat. Mech. Appl..

[10] Ferraz de Arruda H., Nascimento Silva F., Queiroz Marinho V., Raphael Amancio D., da Fontoura Costa L. (2017). Representation of texts as complex networks: A mesoscopic approach. J. Complex Netw..

[11] De Arruda H.F., Marinho V.Q., Costa L.D., Amancio D.R. (2019). Paragraph-based representation of texts: A complex networks approach. Inf. Process. Manag..

[12] Kim J., Wilhelm T. (2008). What is a complex graph?. Phys. A Stat. Mech. Appl..

[13] Van Steen M. (2010). Graph Theory and Complex Networks: An Introduction.

[14] Estrada E. (2012). The Structure of Complex Networks: Theory and Applications.

[15] Gallos L.K., Song C., Makse H.A. (2007). A review of fractality and self-similarity in complex networks. Phys. A Stat. Mech. Appl..

[16] Song C., Havlin S., Makse H.A. (2006). Origins of fractality in the growth of complex networks. Nat. Phys..

[17] Lei M., Liu L., Wei D. (2019). An Improved Method for Measuring the Complexity in Complex Networks Based on Structure Entropy. IEEE Access.

[18] Scott J. (1988). Social network analysis. Sociology.

[19] Song C., Gallos L.K., Havlin S., Makse H.A. (2007). How to calculate the fractal dimension of a complex network: The box covering algorithm. J. Stat. Mech. Theory Exp..

[20] Holme P., Kim B.J., Yoon C.N., Han S.K. (2002). Attack vulnerability of complex networks. Phys. Rev. E Stat. Nonlinear Soft Matter Phys..

[21] Callaway D.S., Newman M.E., Strogatz S.H., Watts D.J. (2000). Network Robustness and Fragility: Percolation on Random Graphs. Phys. Rev. Lett..

[22] Cohen R., Erez K., Ben-Avraham D., Havlin S. (2000). Resilience of the Internet to Random Breakdowns. Phys. Rev. Lett..

[23] Cohen R., Erez K., Ben-Avraham D., Havlin S. (2001). Breakdown of the Internet under Intentional Attack. Phys. Rev. Lett..

[24] Albert R., Jeong H., Barabási A.-L. (2000). Error and attack tolerance of complex networks. Nature.

[25] Gallos L.K., Cohen R., Argyrakis P., Bunde A., Havlin S. (2005). Stability and Topology of Scale-Free Networks under Attack and Defense Strategies. Phys. Rev. Lett..

[26] Gallos L.K., Cohen R., Argyrakis P., Bunde A., Havlin S., Meyers R.A. (2009). Fractal and Transfractal Scale-Free Networks, in Encyclopedia of Complexity and Systems Science.

[27] Iyer S., Killingback T., Sundaram B., Wang Z. (2013). Attack Robustness and Centrality of Complex Networks. PLoS ONE.

[28] Hall M., Frank E., Holmes G., Pfahringer B., Reutemann P., Witten I.H. (2009). The WEKA data mining software: An update. ACM SIGKDD Explor. Newsl..

[29] Hutter F., Hoos H.H., Leyton-Brown K. (2011). Sequential Model-Based Optimisation for General Algorithm Configuration.

[30] Kotthoff L., Thornton C., Hoos H.H., Hutter F., Leyton-Brown K., Hutter F., Kotthoff L., Vanschoren J. (2019). Auto-WEKA: Automatic Model Selection and Hyperparameter Optimization in WEKA. Automated Machine Learning: Methods, Systems, Challenges.

[31] Quinlan J.R. (2014). C4. 5: Programs for Machine Learning.

[32] Ramirez-Arellano A., Bory-Reyes J., Hernandez-Simon L.M. (2018). Statistical Entropy Measures in C4.5 Trees. Int. J. Data Warehous. Min. (IJDWM).

[33] Tharwat A. (2018). Classification assessment methods. Appl. Comput. Inform..

[34] Hand D.J., Till R.J. (2001). A Simple Generalisation of the Area Under the ROC Curve for Multiple Class Classification Problems. Mach. Learn..

[35] Demšar J. (2006). Statistical comparisons of classifiers over multiple data sets. J. Mach. Learn. Res..

[36] Scheirer C.J., Ray W.S., Hare N. (1976). The Analysis of Ranked Data Derived from Completely Randomised Factorial Designs. Biometrics.

[37] Dytham C. (2011). Choosing and Using Statistics: A Biologist’s Guide.

[38] Ennos A.R. (2007). Statistical and Data Handling Skills in Biology.

[39] Akaike H. (1974). A new look at the statistical model identification. IEEE Trans. Autom. Control.

[40] Ramirez-Arellano A. (2019). Students learning pathways in higher blended education: An analysis of complex networks perspective. Comput. Educ..

[41] Burnham K.P., Anderson D.R. (2004). Multimodel Inference: Understanding AIC and BIC in Model Selection. Sociol. Methods Res..

[42] Burnham P.K., Anderson D.R. (2002). Model Selection and Multimodel Inference: A Practical Information-Theoretic Approach.

[43] Andres J. (2010). On a Conjecture about the Fractal Structure of Language. J. Quant. Linguist..

[44] Hrebíček L.K. (1994). Fractals in language. J. Quant. Linguist..

[45] Glattre H.R., Glattre E. (2018). Finding Fractal Networks in Literature. Nonlinear Dyn. Psychol Life Sci..

[46] Kohler R. (1997). Are there fractal structures in language? Units of measurement and dimensions in linguistics. J. Quant. Linguist..

