# Supplementary material for: Classification of Literary Works: Fractality and Complexity of the Narrative, Essay, and Research Article
Source: Entropy (Basel). 2020 Aug 17;22(8):904. doi: 10.3390/e22080904 (PMC7848887; doi:10.3390/e22080904)
Supplement: Supplementary file 1 [file entropy-22-00904-s001.pdf]

Classification of Literary Works: Fractality and Complexity of the Narrative, Essay, and Research Article

SUPPLEMENTARY MATERIAL

The Supplementary Material is organized as follows: Section I present de details of the literacy works included in this article, and Section II shows the details of computation to classify the word co-occurrence network.

I Details of literacy woks

Table S1 shows the title, primary author and the gender of the literacy works included in this study.

Table S1. Title, primary author and the gender of the literacy works.

ID	Title (Spanish)	Primary author	Genre
E1	Carcel de amor	Alfonso Reyes Ocho	Essay
E2	El paisaje en la poesía	Alfonso Reyes Ocho	Essay
E3	Las tres electras	Alfonso Reyes Ocho	Essay
E4	Sobre Góngora	Alfonso Reyes Ocho	Essay
E5	Sobre Mallarmé	Alfonso Reyes Ocho	Essay
E6	Sobre Rimas Bizantinas	Alfonso Reyes Ocho	Essay
E7	Conquista y reconquista del nuevo mundo	Carlos Fuentes Macías	Essay
E8	El siglo de oro	Carlos Fuentes Macías	Essay
E9	Latinoamérica	Carlos Fuentes Macías	Essay
E10	Control y Condón	Carlos Monsiváis Aceves	Essay
E11	Del rancho al internet	Carlos Monsiváis Aceves	Essay
E12	Izquierda Mexicana	Carlos Monsiváis Aceves	Essay
E13	Juan Gabriel	Carlos Monsiváis Aceves	Essay
E14	La crisis, el narcotráfico	Carlos Monsiváis Aceves	Essay
E15	Tú, joven	Carlos Monsiváis Aceves	Essay
E16	El laberinto de la soledad	Octavio Irineo Paz Lozano	Essay
E17	El ogro filantrópico	Octavio Irineo Paz Lozano	Essay
E18	La otra voz	Octavio Irineo Paz Lozano	Essay
E19	Los hijos del limo	Octavio Irineo Paz Lozano	Essay
E20	Máscaras mexicanas	Octavio Irineo Paz Lozano	Essay
E21	Poesía y Tecnología	Octavio Irineo Paz Lozano	Essay
N1	Aura	Carlos Fuentes Macías	Narrative (Novel)
N2	Las batallas en el desierto	José Emilio Pacheco Berny	Narrative (Novel)
N3	Dos Crímenes	Jorge Ibargüengoitia Antillón	Narrative (Novel)
N4	Estas ruinas que ves	Jorge Ibargüengoitia Antillón	Narrative (Novel)

N5	Los relámpaos de Agosto	Jorge Ibargüengoitia Antillón	Narrative (Novel)
N6	La feria	Juan José Arreola Zúñiga	Narrative (Novel)
T1	Chac Mool	Carlos Fuentes Macías	Narrative (Tale)
T2	Un alma pura	Carlos Fuentes Macías	Narrative (Tale)
T3	El principio del placer	José Emilio Pacheco Berny	Narrative (Tale)
T4	La fiesta brava	José Emilio Pacheco Berny	Narrative (Tale)
T5	La zarpa	José Emilio Pacheco Berny	Narrative (Tale)
T6	Langerhaus	José Emilio Pacheco Berny	Narrative (Tale)
T7	Tenga para que se entretenga	José Emilio Pacheco Berny	Narrative (Tale)
T8	Episodio Cinematográfico	Jorge Ibargüengoitia Antillón	Narrative (Tale)
T9	Falta de espíritu scout	Jorge Ibargüengoitia Antillón	Narrative (Tale)
T10	La ley de Herodes	Jorge Ibargüengoitia Antillón	Narrative (Tale)
T11	De balística	Juan José Arreola Zúñiga	Narrative (Tale)
T12	El guardagujas	Juan José Arreola Zúñiga	Narrative (Tale)
T13	El prodigioso miligramo	Juan José Arreola Zúñiga	Narrative (Tale)
T14	Pablo	Juan José Arreola Zúñiga	Narrative (Tale)
T15	Pacto con el diablo	Juan José Arreola Zúñiga	Narrative (Tale)
RA1	La Descripción geográfico-moral del arzobispo Cortés y Larraz (1770) y la historia léxica de Centroamérica: algunos datos salvadoreños	José Luis Ramirez Luengo	Research Article
RA2	Tipos de predicación metonímica en la composición nominal del español de México	Zacarías Ponce de León	Research Article
RA3	Elecciones y Lecciones Poéticas de José Emilio Pacheco	Carmen Alemany Bay	Research Article
RA4	Los clíticos pronominales del español. Estructura y función	Sergio Bogard	Research Article
RA5	Sobre la "imprecisión" de "cierto"	Graciela Fernández Ruiz	Research Article
RA6	Africanismos en el español de México	Luis Fernando Lara	Research Article
RA7	El estatus categorial de "puros" prenominal: un determinante improbable	Violeta Vázquez Rojas Maldonado	Research Article
RA8	Entre la regeneración educativa y las polémicas literarias: la "sátira gramatical" y la figura del	Javier Espino Martín	Research Article

	profesor de latinidad en escritores y "hombres de letras" del siglo XVIII español		
RA9	Deslocativización y abstracción diacrónica de frases preposicionales con a. Efecto dominó en un cambio léxico-semántico	Rodrigo Flores Dávila	Research Article
RA10	Variables en un sistema deíctico binario: aquí, acá, ahí, allí y allá en el español de México	Sara Stradioto	Research Article
RA11	Entonación de los enunciados aseverativos en el español de Tuxtla Gutiérrez, Chiapas	Erika Mendoza Vázquez	Research Article
RA12	La ausencia de concordancia entre el pronombre dativo y su referente nominal en esquemas verbales ditransitivos	Vanina Andrea Barbeito	Research Article
RA13	Rumores y otros malos hábitos. El condicional evidencial en español	Fernando Bermúdez	Research Article
RA14	Creación de un modelo estadístico predictivo para la determinación de las funciones de atenuación en español hablado	Adrián Cabedo Nebot	Research Article
RA15	Enfoque cognitivo para la comprensión de narraciones: una mirada desde la psicología discursiva y el modelo de indexación de eventos	Violeta Cautin-Epifani	Research Article
RA16	Marcas Lingüístico-Discursivas en Titulares de Prensa en Español Actual	Ángel Cervera Rodríguez	Research Article
RA17	Hablando en chiquito -ito en el español de Houston	Manuel J. Gutiérrez	Research Article
RA18	'Se los dije [a ellos]' por 'se lo dije [a ellos]' en el Atlas Lingüístico de México	José G. Moreno de Alba	Research Article
RA19	Expresión de tú genérico y actividades de imagen	Leonor Orozco	Research Article
RA20	El rol de los elementos referenciales en la indexicalidad social	Reisner de Jesús Ravelo Mendez	Research Article
RA21	Actividades de imagen de rol, de autocortesía y de (des)cortesía en reseñas de publicaciones científicas	Silvia Kaul de Marlangeon	Research Article

The corpus in plain text format is available at https://osf.io/t5qnp, the file name corresponds to the column ID of Table S1.

II Fractality of word co-occurrence network

The Akaike Information Criterion (AIC) is applied to classify complex networks as fractal and non-fractal. The AIC for power (denoted by subscript P) and exponential (denoted by subscript E) models — see eqs. **Error! Reference source not found.** and **Error! Reference source not found.** int the main text— is computed, then the min value is chosen (AIC_{min}). The ΔAIC_i was computed by AIC_i - AIC_{min} where *i* is the AIC of power or exponential models. The better model is that have ΔAIC=0 and ΔAIC of the remainder models is greater than two. The adjusted determination coefficient $\overline{R}^2$, AIC, ΔAIC of the networks based on the full text—see Table S2—, by deleting numbers, punctuation marks and functional words —see Table S3—, by adding a lemmatisation stage to the previous ones —see Table S4—, and the fourth one only by a lemmatisation stage —see Table S5.

Table S2. The adjusted determination coefficient $\overline{R}^2$, AIC, ΔAIC and classification for the networks based the full text.

ID	$\overline{R}_P^2$	$\overline{R}_E^2$	AIC_P	AIC_E	ΔAIC_P	ΔAIC_E	Fractal
E1	0.996	0.996	61.2386	64.2371	0	3.00	Yes
E2	0.998	0.998	62.5024	64.1558	0	1.65	Undetermined
E3	0.998	0.998	59.1653	60.9145	0	1.75	Undetermined
E4	0.998	0.998	64.5852	67.0206	0	2.44	Yes
E5	0.997	0.997	90.7550	95.5924	0	4.84	Yes
E6	0.997	0.997	59.4538	62.4514	0	3.00	Yes
E7	0.997	0.997	67.0082	69.2524	0	2.24	Yes
E8	0.997	0.998	56.1585	58.0271	0	1.87	Undetermined
E9	0.997	0.997	62.9739	65.4650	0	2.49	Yes
E10	0.997	0.997	59.5441	62.0833	0	2.54	Yes
E11	0.998	0.998	57.6988	59.2293	0	1.53	Undetermined
E12	0.997	0.997	53.7325	55.6416	0	1.91	Undetermined
E13	0.996	0.996	67.8545	70.6247	0	2.77	Yes
E14	0.997	0.997	56.8002	58.6136	0	1.81	Undetermined
E15	0.998	0.998	62.1102	64.2819	0	2.17	Yes
E16	1.000	0.999	63.6301	64.7574	0	1.13	Undetermined
E17	0.998	0.998	67.7939	70.6391	0	2.85	Yes
E18	0.999	0.999	79.8477	82.8839	0	3.04	Yes
E19	0.999	0.999	91.3729	94.6278	0	3.25	Yes
E20	0.998	0.998	52.5201	54.5671	0	2.05	Yes
E21	0.998	0.998	59.1840	61.2600	0	2.08	Yes
N1	0.999	0.999	69.0498	71.4162	0	2.37	Yes
N2	0.999	0.999	72.5687	75.1390	0	2.57	Yes
N3	1.000	1.000	59.6429	61.9507	0	2.31	Yes
N4	0.999	0.999	60.6780	62.9531	0	2.28	Yes
N5	0.999	0.999	57.7851	60.1895	0	2.40	Yes
N6	0.999	0.999	63.3206	65.6789	0	2.36	Yes
T1	0.997	0.997	58.4204	61.4312	0	3.01	Yes
T2	0.998	0.998	75.0155	78.2709	0	3.26	Yes
T3	0.999	0.999	52.6647	54.6569	0	1.99	Undetermined
T4	0.998	0.998	81.0145	84.3656	0	3.35	Yes
T5	0.996	0.997	50.7199	54.5852	0	3.87	Yes
T6	0.997	0.997	56.8685	60.0643	0	3.20	Yes
T7	0.997	0.997	71.1319	75.8123	0	4.68	Yes
T8	0.993	0.993	48.1537	51.3700	0	3.22	Yes
T9	0.998	0.998	56.2016	59.0056	0	2.80	Yes
T10	0.994	0.995	45.3434	49.2978	0	3.95	Yes
T11	0.995	0.995	57.2059	60.5435	0	3.34	Yes
T12	0.995	0.995	56.2297	59.7141	0	3.48	Yes
T13	0.995	0.996	56.2063	59.6698	0	3.46	Yes
T14	0.996	0.996	57.2374	60.0791	0	2.84	Yes
T15	0.997	0.997	44.8645	48.2072	0	3.34	Yes

RA1	0.998	0.998	74.2022	77.9353	0	3.73	Yes
RA2	0.998	0.998	58.5395	61.3218	0	2.78	Yes
RA3	0.998	0.998	55.6885	57.7098	0	2.02	Yes
RA4	0.998	0.998	79.2112	82.8240	0	3.61	Yes
RA5	0.997	0.997	60.1134	63.2408	0	3.13	Yes
RA6	0.998	0.998	87.3998	90.2956	0	2.90	Yes
RA7	0.998	0.998	77.3236	81.2183	0	3.89	Yes
RA8	0.998	0.998	88.8621	91.6907	0	2.83	Yes
RA9	0.998	0.998	63.1308	66.0162	0	2.89	Yes
RA10	0.998	0.998	59.3461	62.4206	0	3.07	Yes
RA11	0.998	0.998	57.6923	60.9758	0	3.28	Yes
RA12	0.996	0.996	65.5822	69.7744	0	4.19	Yes
RA13	0.997	0.997	67.7274	71.8223	0	4.09	Yes
RA14	0.997	0.997	78.9125	85.3960	0	6.48	Yes
RA15	0.997	0.997	57.5077	61.0455	0	3.54	Yes
RA16	0.998	0.998	64.0546	66.0793	0	2.02	Yes
RA17	0.999	0.999	58.6641	61.6284	0	2.96	Yes
RA18	0.998	0.998	101.6461	107.2518	0	5.61	Yes
RA19	0.998	0.998	74.1753	78.4760	0	4.30	Yes
RA20	0.996	0.996	63.5138	68.5121	0	5.00	Yes
RA21	0.998	0.998	75.2058	79.1835	0	3.98	Yes

Table S3. The adjusted determination coefficient $\overline{R}^2$, AIC, ΔAIC and classification for the networks obtained by deleting numbers, punctuation marks and functional words.

ID	$\overline{R}_P^2$	$\overline{R}_E^2$	AIC$_P$	AIC$_E$	ΔAIC$_P$	ΔAIC$_E$	Fractal
E1	0.988	0.994	165.0796	165.1279	0	0.05	Undetermined
E2	0.992	0.993	147.5352	154.1279	0	6.59	Yes
E3	0.993	0.994	127.0791	134.2273	0	7.15	Yes
E4	0.989	0.993	154.1489	156.3534	0	2.20	Yes
E5	0.991	0.995	184.3288	191.1943	0	6.87	Yes
E6	0.991	0.994	174.1450	185.2053	0	11.06	Yes
E7	0.992	0.994	135.8573	140.4241	0	4.57	Yes
E8	0.993	0.996	152.8502	159.1438	0	6.29	Yes
E9	0.989	0.993	155.7159	154.2666	1.45	0	Undetermined
E10	0.987	0.994	192.0924	192.6042	0	0.51	Undetermined
E11	0.992	0.994	213.5765	223.0195	0	9.44	Yes
E12	0.990	0.993	165.5778	171.6255	0	6.05	Yes
E13	0.990	0.994	171.7393	174.7707	0	3.03	Yes
E14	0.990	0.995	177.2275	180.7225	0	3.50	Yes
E15	0.989	0.994	173.6655	177.5199	0	3.85	Yes
E16	0.995	0.994	137.4156	145.2302	0	7.81	Yes
E17	0.992	0.995	123.6170	125.2777	0	1.66	Undetermined
E18	0.995	0.994	151.9004	162.3274	0	10.43	Yes
E19	0.996	0.995	155.4233	167.5771	0	12.15	Yes
E20	0.991	0.993	121.4777	125.5531	0	4.08	Yes

E21	0.994	0.996	151.9068	163.4284	0	11.52	Yes
N1	0.993	0.993	135.3839	141.8509	0	6.47	Yes
N2	0.994	0.995	217.1279	233.2812	0	16.15	Yes
N3	0.996	0.995	131.1763	139.6924	0	8.52	Yes
N4	0.995	0.994	154.1485	164.5331	0	10.38	Yes
N5	0.995	0.994	134.7868	143.8727	0	9.09	Yes
N6	0.994	0.994	125.7030	134.6883	0	8.99	Yes
T1	0.988	0.994	160.1829	160.7449	0	0.56	Undetermined
T2	0.991	0.995	162.3618	164.6807	0	2.32	Yes
T3	0.994	0.994	139.4552	149.4483	0	9.99	Yes
T4	0.992	0.994	160.4727	166.3170	0	5.84	Yes
T5	0.989	0.994	118.5726	121.8542	0	3.28	Yes
T6	0.990	0.995	186.2044	190.3941	0	4.19	Yes
T7	0.992	0.995	133.8926	145.0800	0	11.19	Yes
T8	0.986	0.995	147.5783	145.9861	1.59	0	Undetermined
T9	0.991	0.995	129.4866	131.6132	0	2.13	Yes
T10	0.989	0.996	130.2684	137.9131	0	7.64	Yes
T11	0.990	0.995	168.3483	178.9648	0	10.62	Yes
T12	0.989	0.993	121.9560	124.5046	0	2.55	Yes
T13	0.985	0.993	159.9897	151.2299	8.76	0	No
T14	0.988	0.992	139.5338	141.9067	0	2.37	Yes
T15	0.992	0.994	109.9967	116.0077	0	6.01	Yes
RA1	0.994	0.994	99.5131	107.1486	0	7.64	Yes
RA2	0.995	0.996	113.8359	121.3203	0	7.48	Yes
RA3	0.993	0.993	108.9364	115.9549	0	7.02	Yes
RA4	0.997	0.996	133.5910	151.3269	0	17.74	Yes
RA5	0.994	0.994	106.3745	113.7152	0	7.34	Yes
RA6	0.994	0.995	138.2657	145.3952	0	7.13	Yes
RA7	0.996	0.995	101.5402	111.7390	0	10.20	Yes
RA8	0.995	0.995	205.5874	222.1510	0	16.56	Yes
RA9	0.996	0.996	113.1292	126.5763	0	13.45	Yes
RA10	0.997	0.996	86.8721	100.1082	0	13.24	Yes
RA11	0.996	0.996	108.3469	119.2230	0	10.88	Yes
RA12	0.996	0.997	186.0979	211.7090	0	25.61	Yes
RA13	0.995	0.995	110.8720	123.4779	0	12.61	Yes
RA14	0.994	0.996	137.5524	146.2809	0	8.73	Yes
RA15	0.994	0.994	95.1961	102.6544	0	7.46	Yes
RA16	0.993	0.994	157.3638	165.4929	0	8.13	Yes
RA17	0.996	0.995	100.0305	110.2642	0	10.23	Yes
RA18	0.995	0.995	152.8813	169.8495	0	16.97	Yes
RA19	0.995	0.995	100.0568	110.0972	0	10.04	Yes
RA20	0.994	0.996	124.7578	131.8060	0	7.05	Yes
RA21	0.994	0.994	104.4961	110.9249	0	6.43	Yes

Table S4. The adjusted determination coefficient $\overline{R}^2$, AIC, ΔAIC and classification for the networks obtained by deleting numbers, punctuation marks, functional words and a lemmatisation stage.

ID	$\overline{R}_P^2$	$\overline{R}_E^2$	AIC$_P$	AIC$_E$	ΔAIC$_P$	ΔAIC$_E$	Fractal
E1	0.988	0.994	152.3427	149.4108	2.93	0	No
E2	0.990	0.992	133.6562	135.1453	0	1.49	Undetermined
E3	0.991	0.991	114.7420	118.1969	0	3.45	Yes
E4	0.988	0.993	159.9204	154.1137	5.81	0	No
E5	0.989	0.994	150.5903	151.3048	0	0.71	Undetermined
E6	0.988	0.993	126.3772	126.8665	0	0.49	Undetermined
E7	0.991	0.993	122.6138	121.9446	0.67	0	Undetermined
E8	0.992	0.994	122.7600	125.9089	0	3.15	Yes
E9	0.989	0.992	106.6197	106.0504	0.57	0	Undetermined
E10	0.988	0.995	204.6782	203.2812	1.40	0	Undetermined
E11	0.988	0.994	176.6637	171.9104	4.75	0	No
E12	0.988	0.993	139.7535	140.4399	0	0.69	Undetermined
E13	0.985	0.993	173.3285	162.5642	10.76	0	No
E14	0.988	0.994	158.5375	156.0183	2.52	0	No
E15	0.986	0.993	157.4146	153.8046	3.61	0	No
E16	0.994	0.993	106.9995	111.6190	0	4.62	Yes
E17	0.987	0.991	115.5617	114.0828	1.48	0	Undetermined
E18	0.991	0.990	106.5358	109.3908	0	2.86	Yes
E19	0.993	0.991	87.3551	90.9726	0	3.62	Yes
E20	0.988	0.992	128.5888	126.7951	1.79	0	Undetermined
E21	0.991	0.993	112.5400	113.5185	0	0.98	Undetermined
N1	0.989	0.992	112.7835	110.0067	2.78	0	No
N2	0.987	0.992	137.2448	133.8150	3.43	0	No
N3	0.994	0.992	93.0037	97.1420	0	4.14	Yes
N4	0.993	0.992	105.0589	108.5556	0	3.50	Yes
N5	0.993	0.993	109.0986	110.6650	0	1.57	Undetermined
N6	0.994	0.993	128.0464	134.2950	0	6.25	Yes
T1	0.987	0.994	164.3519	158.8478	5.50	0	No
T2	0.988	0.993	124.3031	120.9213	3.38	0	No
T3	0.991	0.992	90.1812	92.6700	0	2.49	Yes
T4	0.986	0.991	132.7355	128.9321	3.80	0	No
T5	0.986	0.993	90.1655	87.9663	2.20	0	No
T6	0.986	0.994	111.4835	104.7401	6.74	0	No
T7	0.988	0.993	136.1122	137.3937	0	1.28	Undetermined
T8	0.983	0.995	149.1809	133.8808	15.30	0	No
T9	0.989	0.994	112.3497	110.3657	1.98	0	Undetermined
T10	0.988	0.996	135.9785	137.7701	0	1.79	Undetermined
T11	0.987	0.995	177.1068	167.9358	9.17	0	No
T12	0.986	0.992	120.5325	114.1649	6.37	0	No
T13	0.987	0.992	146.1011	149.3931	0	3.29	Yes
T14	0.988	0.992	111.3463	112.9271	0	1.58	Undetermined
T15	0.990	0.992	80.4797	83.3672	0	2.89	Yes

RA1	0.990	0.992	92.7064	90.6031	2.10	0	No
RA2	0.990	0.991	75.8039	75.5484	0.26	0	Undetermined
RA3	0.989	0.991	126.2178	126.4075	0	0.19	Undetermined
RA4	0.992	0.992	79.2247	81.7646	0	2.54	Yes
RA5	0.990	0.990	91.2602	92.8947	0	1.63	Undetermined
RA6	0.990	0.991	120.4214	121.9146	0	1.49	Undetermined
RA7	0.992	0.993	111.0167	111.8434	0	0.83	Undetermined
RA8	0.991	0.992	118.7297	120.7046	0	1.97	Undetermined
RA9	0.991	0.991	97.7210	99.8927	0	2.17	Yes
RA10	0.992	0.992	73.9521	76.1432	0	2.19	Yes
RA11	0.989	0.991	74.8081	72.1839	2.62	0	No
RA12	0.989	0.992	112.1428	111.7666	0.38	0	Undetermined
RA13	0.990	0.992	98.3513	97.7945	0.56	0	Undetermined
RA14	0.990	0.992	87.9362	87.2881	0.65	0	Undetermined
RA15	0.991	0.992	85.8605	87.9168	0	2.06	Yes
RA16	0.987	0.991	114.7352	111.8029	2.93	0	No
RA17	0.993	0.992	75.0365	80.6909	0	5.65	Yes
RA18	0.989	0.994	115.2253	109.6775	5.55	0	No
RA19	0.990	0.990	84.6207	86.1711	0	1.55	Undetermined
RA20	0.988	0.993	86.0768	80.3460	5.73	0	No
RA21	0.990	0.991	87.1383	87.6202	0	0.48	Undetermined

Table S5. The adjusted determination coefficient $\overline{R}^2$, AIC, ΔAIC and classification for the networks obtained only by a lemmatisation stage.

ID	$\overline{R}_P^2$	$\overline{R}_E^2$	AIC_P	AIC_E	ΔAIC_P	ΔAIC_E	Fractal
E1	0.996	0.996	61.9248	64.8247	0	2.90	Yes
E2	0.998	0.998	62.5739	64.2326	0	1.66	Undetermined
E3	0.998	0.998	59.1856	60.9564	0	1.77	Undetermined
E4	0.998	0.998	64.5667	67.0382	0	2.47	Yes
E5	0.997	0.997	90.8691	95.8550	0	4.99	Yes
E6	0.997	0.997	59.6846	62.7506	0	3.07	Yes
E7	0.997	0.997	67.1121	69.4402	0	2.33	Yes
E8	0.997	0.997	56.2646	58.1463	0	1.88	Undetermined
E9	0.997	0.997	63.2319	65.7886	0	2.56	Yes
E10	0.997	0.997	59.6254	62.1590	0	2.53	Yes
E11	0.998	0.998	57.6536	59.2230	0	1.57	Undetermined
E12	0.997	0.997	53.8671	55.7791	0	1.91	Undetermined
E13	0.996	0.996	67.5798	70.4632	0	2.88	Yes
E14	0.997	0.997	56.7336	58.6194	0	1.89	Undetermined
E15	0.998	0.998	62.2363	64.4159	0	2.18	Yes
E16	1.000	0.999	65.1890	64.8867	0.30	0	Undetermined
E17	0.998	0.998	68.2819	71.1316	0	2.85	Yes
E18	0.999	0.999	80.0278	83.0788	0	3.05	Yes
E19	0.999	0.999	91.9971	95.2262	0	3.23	Yes
E20	0.998	0.998	52.6818	54.7260	0	2.04	Yes

E21	0.998	0.998	59.6121	61.7391	0	2.13	Yes
N1	0.999	0.999	68.9978	71.5149	0	2.52	Yes
N2	0.999	0.999	72.2938	75.0494	0	2.76	Yes
N3	1.000	1.000	59.6131	61.9396	0	2.33	Yes
N4	0.999	0.999	60.9381	63.2042	0	2.27	Yes
N5	0.999	0.999	58.1204	60.5084	0	2.39	Yes
N6	0.999	0.999	63.7196	66.0553	0	2.34	Yes
T1	0.997	0.997	58.1945	61.4387	0	3.24	Yes
T2	0.998	0.998	75.5781	78.8430	0	3.26	Yes
T3	0.999	0.999	52.6986	54.7749	0	2.08	Yes
T4	0.998	0.998	81.3232	84.6822	0	3.36	Yes
T5	0.997	0.997	50.0904	54.2522	0	4.16	Yes
T6	0.997	0.997	56.9160	60.1736	0	3.26	Yes
T7	0.997	0.997	71.5703	76.2529	0	4.68	Yes
T8	0.992	0.993	48.5365	51.6863	0	3.15	Yes
T9	0.997	0.997	57.0120	59.7341	0	2.72	Yes
T10	0.995	0.995	45.1567	49.4237	0	4.27	Yes
T11	0.995	0.995	57.5544	60.8403	0	3.29	Yes
T12	0.994	0.994	57.1215	60.5362	0	3.41	Yes
T13	0.995	0.996	56.2252	59.7875	0	3.56	Yes
T14	0.996	0.996	57.3605	60.2562	0	2.90	Yes
T15	0.997	0.997	45.7179	48.9596	0	3.24	Yes
RA1	0.998	0.998	74.6321	78.3600	0	3.73	Yes
RA2	0.998	0.998	58.5891	61.3572	0	2.77	Yes
RA3	0.998	0.998	55.9569	57.9578	0	2.00	Yes
RA4	0.998	0.998	79.8479	83.4438	0	3.60	Yes
RA5	0.997	0.997	60.2851	63.5158	0	3.23	Yes
RA6	0.998	0.998	96.2085	99.6156	0	3.41	Yes
RA7	0.998	0.998	77.1624	81.1508	0	3.99	Yes
RA8	0.998	0.998	88.7850	91.6958	0	2.91	Yes
RA9	0.998	0.998	63.2926	66.2575	0	2.96	Yes
RA10	0.998	0.998	59.6712	62.8164	0	3.15	Yes
RA11	0.998	0.998	64.7880	68.6146	0	3.83	Yes
RA12	0.996	0.996	66.6305	70.8120	0	4.18	Yes
RA13	0.997	0.997	67.8030	71.9992	0	4.20	Yes
RA14	0.997	0.997	79.1745	85.7515	0	6.58	Yes
RA15	0.997	0.997	57.7785	61.4855	0	3.71	Yes
RA16	0.998	0.998	64.6109	66.6818	0	2.07	Yes
RA17	0.999	0.999	58.6381	61.7309	0	3.09	Yes
RA18	0.998	0.998	102.5060	108.1882	0	5.68	Yes
RA19	0.998	0.998	75.4587	79.3705	0	3.91	Yes
RA20	0.996	0.996	62.7674	68.2808	0	5.51	Yes
RA21	0.998	0.998	75.4492	79.4704	0	4.02	Yes
